# Psilocybin-assisted therapy for treatment-resistant depression in the US: a model-based cost-effectiveness analysis

**DOI:** 10.1038/s41398-025-03556-4

**Published:** 2025-08-29

**Authors:** Anton L. V. Avanceña, Linh Vuong, James G. Kahn, Elliot Marseille

**Affiliations:** 1 Health Outcomes Division, College of Pharmacy, The University of Texas at Austin, Austin, TX USA; 2 Department of Internal Medicine, Dell Medical School, The University of Texas at Austin, Austin, TX USA; 3 College of Pharmacy, The University of Texas at Austin, Austin, TX USA; 4 Collaborative for the Economics of Psychedelics (CEP), UC Berkeley/UC San Francisco Center for Global Health Delivery, Diplomacy, and Economics, Berkeley, CA USA; 5 Philip R. Lee Institute for Health Policy Studies, School of Medicine, UC San Francisco, San Francisco, CA USA; 6 Department of Epidemiology and Biostatistics, School of Medicine, UC San Francisco, San Francisco, CA USA; 7 Health Strategies International, Oakland, CA USA

**Keywords:** Depression, Psychiatric disorders

## Abstract

Psilocybin-assisted therapy (PAT) has been shown in early trials to reduce the symptoms of treatment-resistant depression (TRD). This study evaluated the cost-effectiveness of PAT as a third-line treatment for major depressive disorder compared to standard of care (SOC). We used an individual-level, probabilistic simulation model that portrays representative US adults with TRD who receive SOC (pharmacotherapy, psychotherapy, electroconvulsive therapy, and esketamine nasal spray) and PAT over 12 months. We assumed the total cost of PAT was $5000, which we varied in sensitivity analyses ($3000–20,000). We calculated total costs, health effects (in terms of quality-adjusted life years [QALYs] gained), and incremental cost-effectiveness ratio (ICER) from limited healthcare and societal perspectives. PAT leads to an additional 0.031 QALYs and $3639 costs compared to SOC over 12 months, giving an ICER of $117,517 per QALY gained from a limited healthcare perspective. Using a $150,000 cost-effectiveness threshold, PAT had a 75% probability of being the cost-effective choice, and it was associated with a lower expected loss than SOC ($301 vs. $1307). Results were sensitive to uncertainty in model parameters, particularly the cost of PAT. PAT had a 1% probability of being cost-effective when its overall costs were $10,000 and 95% when its costs were $3000. This cost-effectiveness analysis found that when its costs are $5000 or less, PAT may offer economic value compared to available TRD treatments. Future studies can explore ways to reduce the cost of PAT and to understand its long-term effectiveness in maintaining remission and reducing the risk of relapse.

## Introduction

Major depressive disorder (MDD) is the most common mood disorder globally [1]. In the US, an estimated 21 million adults or 8.3% of the adult population have MDD [2], and the annual health, social, and economic costs total $330 billion [3]. While several pharmacological and psychosocial interventions are available to treat MDD [4, 5], about one-third of people with MDD do not adequately respond, a condition called treatment-resistant depression (TRD) [6]. TRD is associated with higher all-cause and suicide-specific mortality [7, 8] and higher overall and mental health-related costs than responsive MDD [6, 9–11].

In an effort to develop new MDD treatments, psychedelic therapies have been tested in randomized clinical trials (RCTs) for their efficacy in reducing MDD symptoms [12, 13]. Among the most studied psychedelics is psilocybin, a naturally occurring serotonin 1A/2A receptor agonist found in *Psilocybe* fungi. A 6-week RCT (*N* = 104) found that 25% of participants with MDD who received a single-dose psilocybin-assisted therapy (PAT) achieved sustained remission, compared to 9.1% in the active placebo group (*P* < 0.05) [14]. An earlier and smaller RCT among individuals with MDD (*N* = 52) found similar results, with a 2-week remission rate of 54% compared to 12% in the control group (*P* < 0.01) [15]. Finally, in an RCT focused on individuals with TRD (*N* = 79), single-dose PAT led to a three-week remission rate of 29% [16], which is higher than the 13.7% remission rate for standard third-line antidepressants [17].

Though PAT may provide relief to people with MDD and TRD, questions remain about its economic value. For instance, PAT requires more initial resources than conventional treatments; trials of PAT included 4–7 days of preparation, active dosing, and integration sessions with trained counselors and therapists [14–16]. During the administration of psilocybin, two trained professionals guide the participant and monitor their response, as required by the Food and Drug Administration (FDA; [18]. By contrast, most antidepressants are not administered by a professional, and psychotherapy typically involves one-hour sessions with one therapist, with treatment length varying from weeks to years depending on a patient’s needs [19]. The efficacy of PAT has also not been directly compared to available TRD treatments, though one RCT did compare PAT to escitalopram, a widely used first-line pharmacotherapy, in a study that focused on non-TRD (i.e., responsive) MDD [20, 21].

In this study, we conducted an exploratory model-based economic evaluation to estimate the costs, health effects, and cost-effectiveness of single-dose PAT compared to standard treatments for TRD. We also identified the determinants of PAT’s overall value, such as the cost of treatment or minimum level of effectiveness, that would allow PAT to meet commonly used criteria for cost-effective health technologies in the US. While a similar analysis has been done in the UK [22], the present study is the first to explore the economics of PAT in the US context. Findings from this study can inform the design of current and future PAT trials and, if approved, pricing and coverage decisions for PAT.

## Methods

### Overview

This study followed guidelines for economic evaluations in health (S1 CHEERS Checklist) [23, 24]. We used a decision-analytic simulation model to estimate the costs, health effects, and cost-effectiveness of single-dose PAT for adults (18 years and older) with TRD. We compared PAT to a set of recommended third-line treatments for MDD, referred to as “standard of care” (SOC), which were weighted by their level of use in the population [4, 17]. Over 99% of individuals in the SOC group receive 2nd generation antidepressants (i.e., pharmacotherapy), which are often used in conjunction with psychotherapy (Table A1 in S2 Appendix). Based on available data [25, 26], we assumed that <1% of individuals in SOC each receive electroconvulsive therapy (ECT) and esketamine nasal spray (Spravato®), which was approved by the FDA for TRD in 2019 and is used in combination with pharmacotherapy (Table 1). In our analysis, we assumed that individuals who do not respond or relapse after receiving ECT, esketamine, or PAT switch to pharmacotherapy for their fourth-line and succeeding therapies.

**Table 1:** Model inputs and parameters.

Input	Base value (SD)	Range^a^	Distribution in probabilistic analysis	Source
Third-line weekly probabilities				
Pharmacotherapy and psychotherapy^b^				
MDD to remission	0.026 (0.0013)	0.0234–0.0286	Beta	Rush et al. [17]
MDD to response	0.0049 (0.0003)	0.0044–0.0054	Beta	Rush et al. [17]
Remission to MDD	0.0353 (0.0018)	0.0318–0.0388	Beta	Rush et al. [17]
Response to MDD	0.1121 (0.0057)	0.1009–0.1233	Beta	Rush et al. [17]
ECT				
MDD to remission	0.1629 (0.0083)	0.1466–0.1792	Beta	Dierckx et al. [32]
MDD to response	0.0418 (0.0021)	0.0376–0.046	Beta	Dierckx et al. [32]
Remission to MDD	0.045 (0.0023)	0.0405–0.0495	Beta	Ross et al. [27]
Response to MDD	0.1634 (0.0083)	0.147–0.1797	Beta	Same as pharmacotherapy and psychotherapy
Esketamine				
MDD to remission	0.1402 (0.0072)	0.1261–0.1542	Beta	Fedgchin et al. [33], Popova et al. [34]
MDD to response	0.0441 (0.0022)	0.0397–0.0485	Beta	Fedgchin et al. [33], Popova et al. [34]
Remission to MDD	0.018 (0.0009)	0.0162–0.0198	Beta	Daly et al. [92]
Response to MDD	0.0796 (0.0041)	0.0716–0.0876	Beta	Daly et al. [92]
PAT				
MDD to remission	0.1079 (0.0055)	0.0971–0.1187	Beta	Goodwin et al. [16]
MDD to response	0.0274 (0.0014)	0.0247–0.0302	Beta	Goodwin et al. [16]
Remission to MDD	0.0353 (0.0018)	0.0318–0.0388	Beta	Same as pharmacotherapy and psychotherapy
Response to MDD	0.1121 (0.0057)	0.1009–0.1233	Beta	Same as pharmacotherapy and psychotherapy
Fourth-line weekly transition probabilities^c^				
MDD to remission	0.0186 (0.001)	0.0168–0.0205	Beta	Rush et al. [17]
MDD to response	0.0047 (0.0002)	0.0042–0.0051	Beta	Rush et al. [17]
Remission to MDD	0.067 (0.0034)	0.0603–0.0737	Beta	Rush et al. [17]
Response to MDD	0.12 (0.0061)	0.108–0.132	Beta	Rush et al. [17]
Response to remission^d^	0.0027 (0.0001)	0.0025–0.003	Beta	Hermens et al. [43], Meeks et al. [40], Richards et al. [41], Wagner et al. [42], Whiteford et al. [44]
Remission to response^d^	0.0013 (0.0001)	0.0011–0.0014	Beta	Judd et al. [39], Sobocki et al. [38]
Total cost of third-line MDD treatment (US$)				
Pharmacotherapy and psychotherapy^c^	36 (4.59)	27–45	Gamma	2018 [47]
ECT	5409 (690)	4057–6761	Gamma	Agbese et al. [48], Ross et al. [27]
Esketamine	5882 (750)	4412–7353	Gamma	Brendle et al. [35]
PAT	5000 (NA)	3000–20000	NA	Assumed by authors^e^
Uptake of third-line SOC therapies				
Pharmacotherapy and psychotherapy	99.59%	NA	NA	Calculated by authors^f^
ECT	0.25%	NA	NA	Calculated by authors^f^
Esketamine	0.16%	NA	NA	Calculated by authors^f^
Weekly costs (US$)^g^				
Maintenance and continuation treatment^h^				
ECT	225 (29)	169–282	Gamma	Agbese et al. [48], Ross et al. [27]
Esketamine	1059 (135)	794–1324	Gamma	Brendle et al. [35]
Other healthcare				
MDD	174 (22)	130–217	Gamma	Amos et al. [47]
Response	118 (15)	89–148	Gamma	Amos et al. [47]
Productivity^i^	155 (36)	84–226	Gamma	Zhdanava et al. [6]
Health utilities^j^				
MDD	0.5 (0.038)	0.43–0.58	Beta	Balázs et al. [54]
Response	0.75 (0.043)	0.67–0.84	Beta	Balázs et al. [54]
Remission	0.82 (0.048)	0.72–0.91	Beta	Balázs et al. [54]

*ECT* electroconvulsive therapy, *MDD* major depressive disorder, *NA* not applicable, *PAT* psilocybin-assisted therapy, *SD* standard deviation.

^a^Where available, ranges and SDs were taken from the original reference or source. Otherwise, authors applied ±25% to the base value to construct a range, and an SD was estimated by dividing the range by 4.

^b^“Pharmacotherapy and psychotherapy” includes the set of recommended step therapy for MDD. These treatments, together with ECT and esketamine, constitute the “standard of care” for TRD to which PAT is being compared.

^c^In this study, individuals who fail third-line therapy receive pharmacotherapy for their fourth-line and succeeding therapies.

^d^These probabilities apply to all lines of therapy.

^e^The cost of PAT has not been established and was deterministically varied by the authors to understand how changes in the cost of PAT affects its economic value as a third-line therapy for MDD.

^f^Calculated by the authors based on previously reported estimates of treatment uptake. See Table A1 in S2 Appendix.

^g^Additional cost inputs are found in Table A3 in S2 Appendix.

^h^Maintenance treatment refers to additional cycles of therapy that are provided to individuals who achieve remission and/or response after the initial treatment.

^i^These productivity costs include the absenteeism and presenteeism of TRD.

^j^These annual health utilities were transformed to weekly utilities to match the cycle length in the model.

We used limited healthcare and societal perspectives (Table A2 in S2 Appendix). Due to the lack of data on the long-term efficacy of PAT, we limited our analysis to a 12-month horizon, and outcomes were not discounted. Analyses were conducted in TreeAge Pro 2025 (TreeAge Software Inc., Williamstown, MA).

### Model

**Fig. 1:**
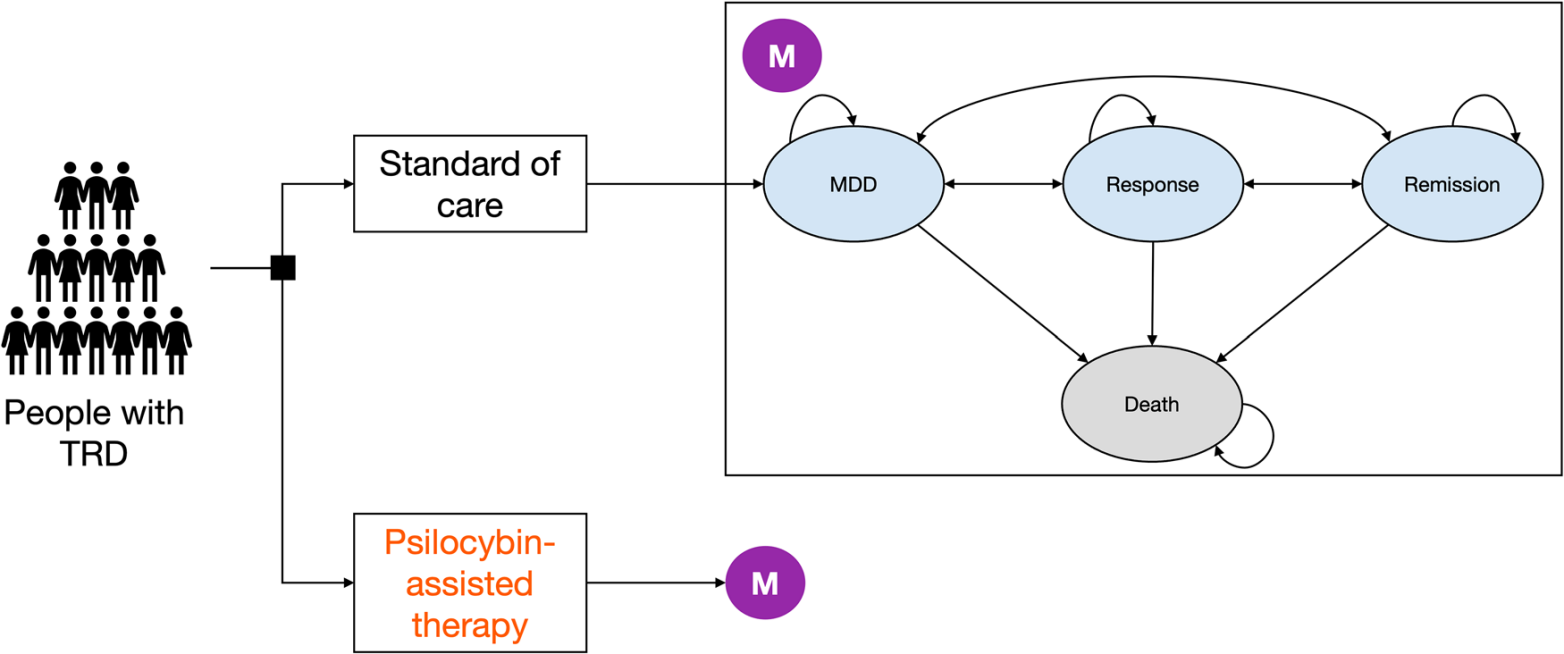
Schematic of the simulation model. This figure shows the structure of the decision-analytic model used in the study. The root of the model shows the two decision alternatives, standard of care and psilocybin-assisted therapy. The purple circle denotes the common Markov node, and ovals are the mutually exclusive health states that are each associated with a cost and health utility. Each arrow represents possible transitions that individuals may experience and is associated with a weekly probability. MDD major depressive disorder.

We developed a probabilistic, individual-level simulation model to conduct this study (Fig. 1). We simulated a representative cohort of individuals with MDD in the US using data from the 2021 National Survey on Drug Use and Health [2]. As in previous analyses [27] and following consultations with psychiatrists, our model included four mutually exclusive health states: MDD, response, remission, and death (Fig. 1). Individuals in our model have TRD and start in the MDD state, and they can progress to other health states based on weekly transition probabilities. We defined TRD based on failure to respond to two MDD treatments—a common but not universal definition of TRD [28]. The model tracks the types and lines of therapy individuals receive over time, and we assumed that all individuals receive treatment. Individuals who experience treatment failure receive the next line of therapy after 8 weeks.

Individuals whose MDD symptoms are completely or nearly abated move to the remission state, which is the primary goal of MDD treatment [29, 30]. In MDD trials and other studies, remission is defined by a sufficiently low score on an MDD scale (e.g., <10 points on the Montgomery-Åsberg Depression Rating Scale). Individuals who experience some reduction in MDD symptoms but do not meet the criteria for remission enter the response state. This definition departs from the common operationalization in RCTs, where response is defined based on a 50% reduction in an individual’s baseline MDD score following treatment. However, this definition can encompass individuals who achieve remission, as well as those who achieve a smaller change in their MDD severity. Because response is a distinct health state in our model, we excluded, where possible, the number of remitters from the number of responders when estimating the probability of response from our primary sources.

### Data and sources

#### Transition probabilities

We estimated weekly transition probabilities (represented by arrows in Fig. 1) from the published literature (Table 1). Probabilities based on other intervals were transformed to weekly intervals using standard procedures [31].

We took the probability of remission and response for each treatment from trials and meta-analytic studies. For pharmacological antidepressants and psychosocial treatment, we used results from the Sequenced Treatment Alternatives to Relieve Depression (STAR*D) study to estimate the probability of response and remission [17]. For ECT, we used rates of remission and response from a meta-analysis [32]. When coupled with antidepressants, esketamine has remission and response rates that are about 30% higher than standard treatments based on prior trials [33–36]. For PAT, we relied on a phase 2 RCT that administered three different doses of psilocybin to randomized groups. That trial found that a single, 25-mg dose of psilocybin coupled with intensive psychosocial support was associated with the most pronounced antidepressant effects, with a remission rate of 29% and a response rate of 8% [16].

We estimated three relapse probabilities from the literature (Table 1). We relied on data from prior trials and observational studies to estimate the probability of MDD given remission and response for pharmacotherapy, ECT, and esketamine [17, 27, 33]. Because the rate of relapse for PAT has not been extensively studied, we assumed that individuals who receive PAT as their third-line therapy have the same relapse rates as third-line therapies in STAR*D [17]. This is likely an overestimate, since follow-up data from a prior PAT trial showed a 12-month 58% remission rate among individuals who received two high doses of PAT [17, 37]. For the probability of response given remission, which represents a worsening of symptoms, we took the average from previous trials and observational studies involving antidepressants, and used the same value for all treatments considered in this analysis [38, 39].

Individuals in our model can also transition from response to remission, which represents an improvement in MDD symptoms. For this probability, we used the average estimate from prior observational and interventional studies [40–44]. Finally, for the probability of death, we took age- and sex-specific data from the 2020 life tables [45]. We incorporated the higher risk of all-cause mortality among people with MDD by multiplying the probability of death with the relative risk of mortality reported in a systematic review [46].

#### Costs

We included different costs in each perspective (Table A2 in S2 Appendix). For the limited healthcare perspective, we included the costs of treatment and all-cause healthcare utilization such as pharmacy, outpatient (including psychotherapy sessions that might be offered with pharmacotherapy, ECT, or esketamine), inpatient, and emergency services. We used data from a previous analysis of a large administrative claims dataset in the US that reported the costs of non-TRD and TRD across different cost categories [47]. For the cost of initial and maintenance ECT and esketamine, we relied on previous cost analyses of these two treatments [27, 35, 48]. All historical costs were updated to 2022 US$ using the medical component of the Consumer Price Index [49].

Based on a recent costing study of PAT for MDD [50], we assumed that the total cost of PAT was $5000 in the base case ($3500 for therapists and $1500 for psilocybin). Because the cost of psilocybin following potential FDA approval is unknown, we deterministically varied the cost of PAT from $3000–$20,000 per treatment. Using a wide range allowed us to understand how changes in the cost of PAT affected its economic value as a third-line therapy for MDD.

For the limited societal perspective, we added the productivity costs of TRD to the costs included in the limited healthcare perspective (Table A3 in S2 Appendix). Productivity costs measure the economic burden of an illness or health condition on labor efficiency and includes absenteeism (missed days of work) and presenteeism (reduced output or functionality at work) [51]. We relied on a previous study of the economic burden of MDD to estimate the incremental productivity cost of TRD [6]. To ensure that costs from this study reflect current economic conditions, we adjusted these costs by the change in full-time earnings reported by US Bureau of Labor Statistics between 2018 and 2022 [52].

#### Health utilities

We measured health outcomes in terms of quality-adjusted life years (QALYs) in this study. A common measure of health benefit in economic evaluations, QALYs represent a year that a person is alive weighted by that person’s health‐related quality of life [53]. QALYs typically have a value of 0 (death) to 1 (a year in perfect health), and preference-based health utilities are used to calculate QALYs for health states between perfect health and death. To estimate QALYs, we relied on a systematic review of health utilities for MDD across different levels of severity that were measured using the time-tradeoff approach [54].

### Analysis

Given the uncertainty in our model parameters, we used probabilistic analyses to estimate mean cost-effectiveness results and understand the effect of parameter uncertainty on our findings [55]. This approach, preferred by some health technology assessment organizations over a deterministic analysis, explicitly quantifies and emphasizes the degree of decision uncertainty [56].

To conduct our probabilistic analysis, we ran our model 1000 times, each with 1000 simulated individuals with MDD. In each model run, a random sample of values for each model input (i.e., transition probabilities, costs, and health utilities) were drawn from distributions assigned to each parameter (Table 1). We then calculated the mean costs and health outcomes and constructed a 95% predicted interval. We also calculated the expected incremental cost-effectiveness ratio (ICER), which is the ratio of incremental mean costs and incremental mean health effects between PAT and SOC. The ICER represents the cost per additional unit of health outcome gained and is compared to a context-specific cost-effectiveness threshold to determine whether an intervention offers economic value. In this study, we used a cost-effectiveness threshold range of $100,000–150,000 per QALY gained, which is typically used in US-based cost-effectiveness analyses [57].

Because we assigned probability distributions to each of our parameters, we conducted conditional probabilistic analyses in lieu of conventional one- and two-way sensitivity analyses [58]. In conditional probabilistic analysis, the value of one or more parameters are deterministically changed while all other parameters are modeled probabilistically. In this study, we focused on varying three inputs: probability of remission, probability of relapse, and cost of PAT. These inputs are the most uncertain yet policy-relevant parameters that will affect PAT’s economic value if approved [50, 59]. For the two probabilities, which represent the effectiveness of PAT, we applied 5, 10, and 15% increases and decreases to their base value, while we varied the cost of PAT from $3000–$10,000. For each combination, we determined the probability that PAT would be the cost-effective option over SOC from limited healthcare and societal perspectives using a cost-effectiveness threshold of $150,000 per QALY gained.

Following guidelines for cost-effectiveness analyses [55, 60], we further summarized the results of the probabilistic analyses in two ways. Using cost-effectiveness acceptability curves, we plotted the proportion of model runs for which each intervention was the cost-effective option against a range of cost-effectiveness thresholds. We also generated expected loss curves, which plot the economic cost or foregone benefits of choosing the suboptimal (i.e., less cost-effective) intervention across different cost-effectiveness thresholds [61, 62]. The intervention with the lowest expected loss is the optimal choice, and the lowest expected loss represents the “expected value of perfect information,” a common metric used to estimate the cost of uncertainty and the value of additional research that can eliminate uncertainty in available evidence [63].

## Results

Assuming a $5000 cost of PAT, the incremental mean cost of PAT was $3638 from a healthcare perspective (Table 2). With a 9% higher overall remission rate, the incremental mean benefit of PAT was 0.031 QALYs gained over 12 months, which is equivalent to 31 QALYs for 1000 individuals with TRD. From a limited healthcare perspective, the expected ICER of PAT was $117,517. The expected ICER was slightly lower (more favorable) from a limited societal perspective at $111,069.

**Table 2:** Mean costs, health effects, and ICERs of PAT using limited healthcare and societal perspectives.

Perspective	Intervention	Mean cost in US$ (95% PI)^a^	Mean QALYs (95% PI)	Expected ICER^b^
Healthcare	Standard of care	11,061 (10.162–11915)	0.540 (0.457–0.620)	
	Psilocybin-assisted therapy	14,700 (13.914–15.474)	0.571 (0.489–0.649)	117,517
Societal	Standard of care	12,564 (11.572–13554)	0.540 (0.457–0.620)	
	Psilocybin-assisted therapy	16,003 (15.090–16.896)	0.571 (0.489–0.649)	111,069

The 95% predicted intervals (PI) represent the 5 and 95th percentile of the 1000 probabilistic simulations conducted. Mean costs were rounded to the nearest dollar, and mean QALYs were rounded to three decimal places.

*ICER* incremental cost-effectiveness ratio, *PI* predicted interval, *QALY* quality-adjusted life year.

^a^The assumed cost of psilocybin-assisted therapy is $5000 per treatment.

^b^ICERs portray the incremental cost per QALY gained and were calculated by comparing psilocybin-assisted therapy to standard of care. ICERs presented here were calculated using unrounded mean costs and QALYs and may therefore result in some discrepancy.

**Fig. 2:**
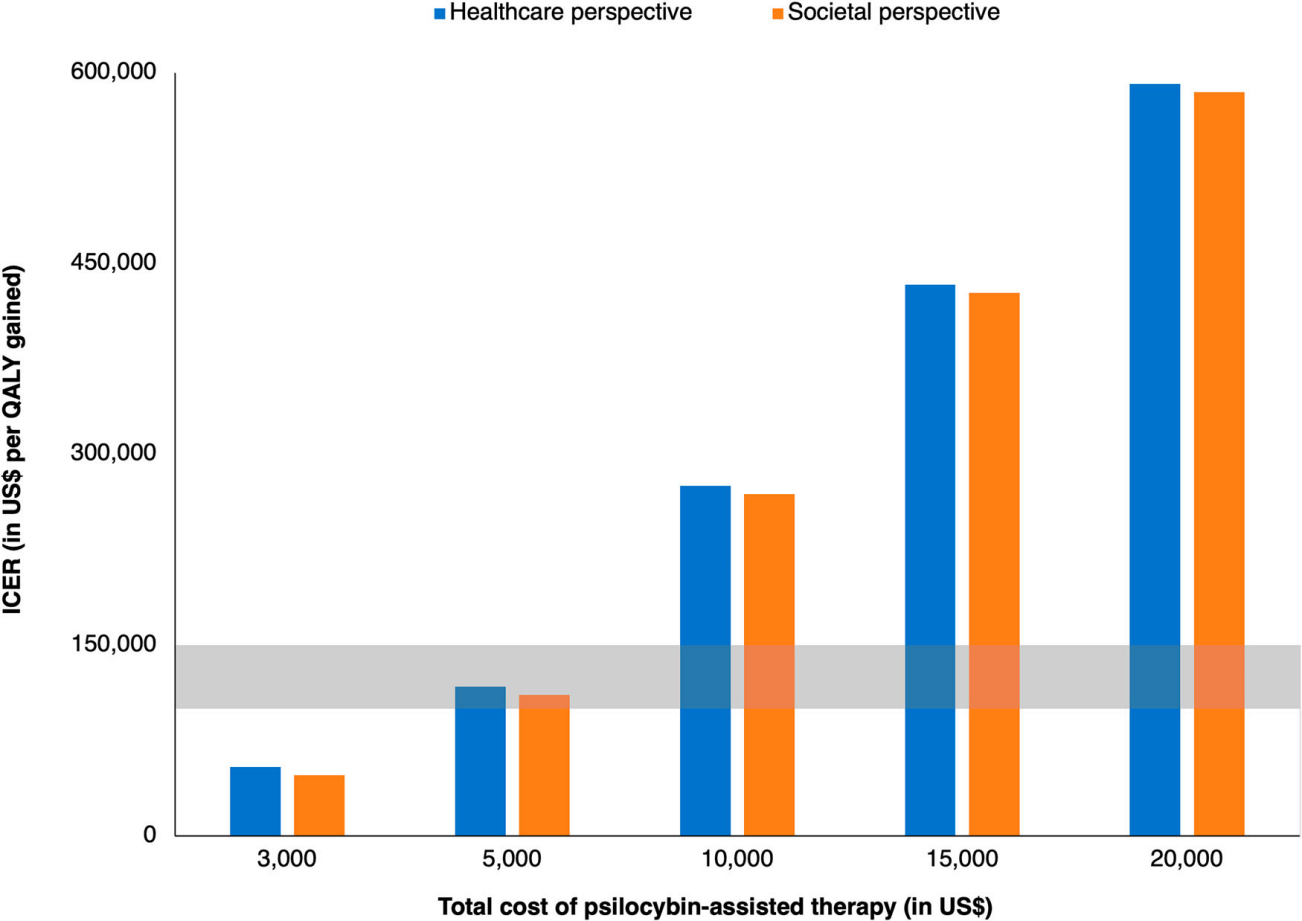
Sensitivity analysis varying the cost of PAT. The vertical bars represent the expected incremental cost-effectiveness ratio of psilocybin-assisted therapy from limited healthcare and societal perspectives. The gray horizontal band represents the conventional cost-effectiveness threshold range used in the US ($100,000–150,000 per QALY gained). ICER incremental cost-effectiveness ratio, QALY quality-adjusted life year.

The sensitivity of the results to the cost of PAT are shown in Fig. 2. PAT met common thresholds for cost-effectiveness (gray band in Fig. 2) when its cost was $5000 or less. At $10,000 per treatment, PAT had higher expected ICERs of $275,426 and $268,978 per QALY gained from limited healthcare and societal perspectives, respectively.

**Fig. 3:**
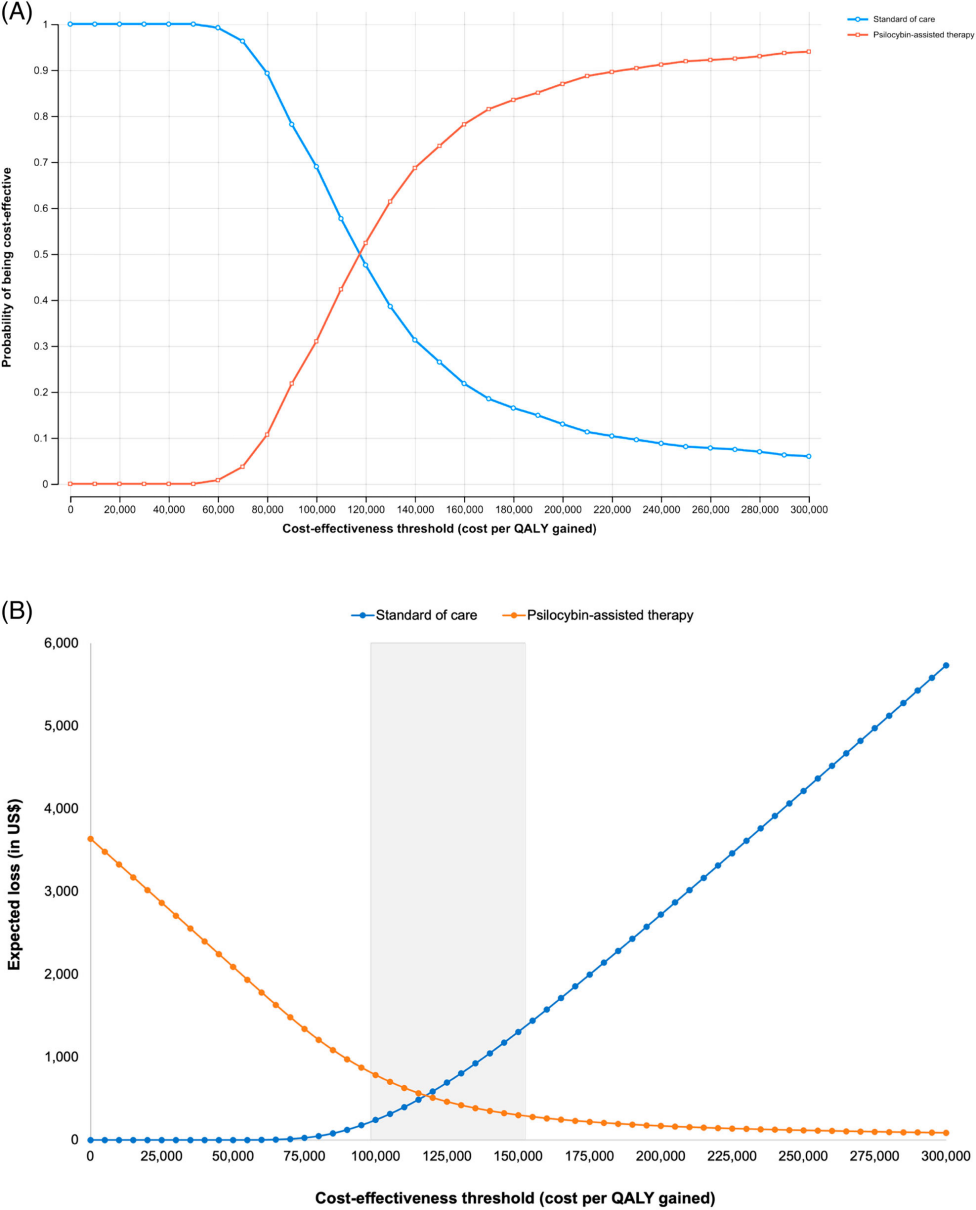
Results of probabilistic sensitivity analysis using a limited healthcare perspective and $5000 cost of PAT. The graphs in this figure summarize the results of the probabilistic analyses from a limited healthcare perspective and assuming a $5000 cost of PAT. Cost-effectiveness acceptability curves (**A**) display the probability that each intervention is cost-effective over a range of cost-effectiveness thresholds. The probabilities are calculated based on the results of the 1000 simulations. Expected loss curves (**B**) plot the per-person cost (or forgone benefits) of choosing an intervention if it is suboptimal. The intervention with the lowest expected loss at any cost-effectiveness threshold is the optimal choice. The cost-effectiveness threshold where the curves cross is equal to the expected incremental cost-effectiveness ratio of PAT. The gray horizontal band represents the conventional cost-effectiveness threshold range used in the US ($100,000–150,000 cost per QALY gained). PAT psilocybin-assisted therapy, QALY quality-adjusted life year.

Figure 3 summarizes the results of the uncertainty analysis assuming $5000 for the cost of PAT from a healthcare perspective. The probability that PAT is cost-effective increases from 31–74% when the cost-effectiveness threshold increases from $100,000–$150,000 (Fig. 3A). With a $150,000 cost-effectiveness threshold, the expected loss of PAT is 77% lower than SOC ($301 vs. $1307 per person), meaning that PAT, with some uncertainty, is the optimal choice at that threshold (Fig. 3B).

When the cost of PAT is $10,000, the probability that it is cost-effective at the $150,000 threshold is 1%, and its expected loss is $3889 compared to $6 for SOC (Fig. A1 in S2 Appendix). Additional sensitivity analysis results under different assumptions are shown in Figs. A2–A6 in S2 Appendix.

**Fig. 4:**
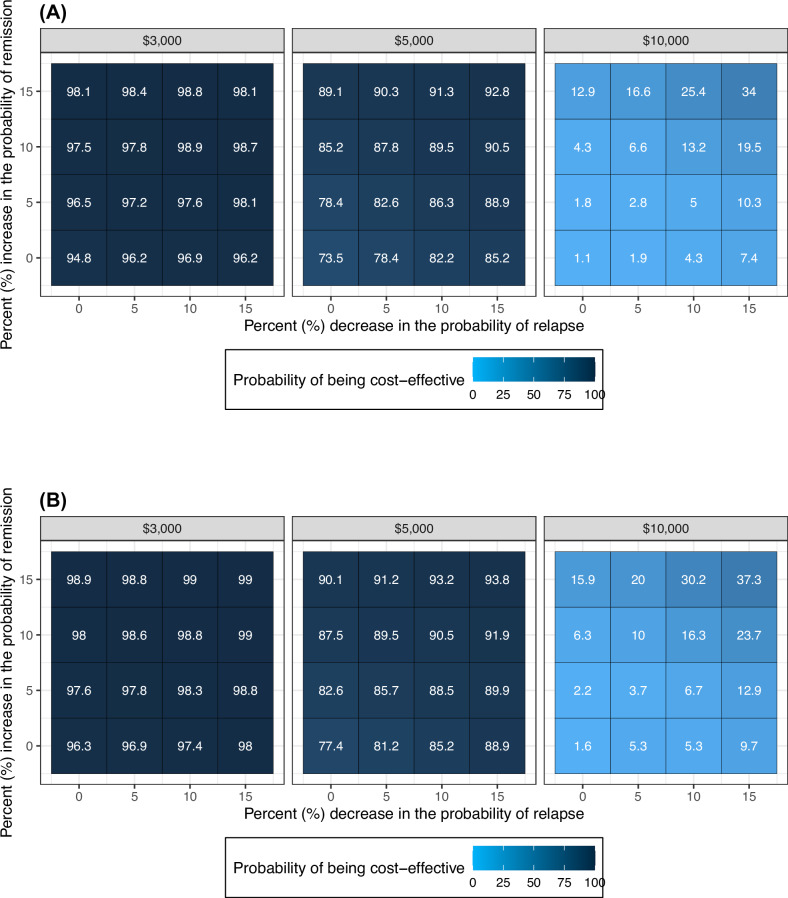
Results of the conditional probabilistic sensitivity analysis. These tile graphs summarize the results of the conditional probabilistic sensitivity analyses from a limited healthcare (**A**) and limited societal (**B**) perspectives. In conditional probabilistic analysis, the values of selected inputs are varied deterministically while all other inputs are modeled probabilistically. The numbers in each tile are the probability that PAT is cost-effective compared to SOC given different values of the probability of remission, probability of relapse, and cost of PAT assuming a $150,000 cost-effectiveness threshold. PAT psilocybin-assisted therapy, SOC standard of care.

Figure 4 shows the results of the conditional probabilistic sensitivity analyses. Changing the probability of remission and probability of relapse of PAT affects its probability of being cost-effective compared to SOC. Increases in the probability of remission (from no change to 15%) have a slightly more pronounced influence on the economic value of PAT than decreases in the probability of relapse. For example, increasing the probability of remission by 15% increases the probability that PAT is cost-effective from 74% to 89% from a limited healthcare perspective when its costs are assumed to be $5000 (Fig. 4A). By contrast, when the probability of relapse is reduced by 15%, PAT’s probability of being cost-effective changes from 74% to 85%.

When the probability of remission and probability of relapse improve simultaneously, the economic value of PAT improves considerably. For example, assuming the cost of PAT is $10,000, the probability that PAT is cost-effective increases from 1% to 34% when the probability of remission increases by 15% and the probability of relapse decreases by 15% (Fig. 4A). We found similar trends under a limited societal perspective (Fig. 4B).

## Discussion

This model-based economic evaluation found that one-time, single-dose PAT can be a cost-effective TRD treatment compared to standard third-line MDD treatments under specific conditions. At $5000 per treatment, PAT was most likely to be the cost-effective intervention and was associated with a lower expected loss than SOC. With higher (e.g., $10,000) costs, PAT is not likely to be cost-effective, even when its effectiveness is higher than what has been reported in previous trials.

Only one other study, to our knowledge, has explored the cost-effectiveness of PAT for MDD. McCrone et al. used a deterministic decision-tree model to compare two-dose PAT with pharmacotherapy, psychotherapy, or combination therapies among UK individuals with “hard to treat depression” [22]. Like the present study, McCrone et al. found that while PAT was associated with improved health (an additional 0.023 QALYs) over 6 months, it did not meet cost-effectiveness thresholds in the UK due to its high incremental costs from a healthcare perspective. (From a societal perspective, PAT was cost-effective when the total costs of PAT were £6132.) Their analysis found that a 50% reduction in the cost of therapist support or a 50% increase in the remission rate would make PAT a cost-effective option.

Despite the similar findings, our analysis departs from the McCrone study in several ways. First, we use an individual-level state-transition model that better portrays sequential and recurring events, such as the progression of MDD, as well as individual characteristics such as age and types and lines of therapy over time. Second, we used a longer time horizon of 12 months and used a more defined population of interest (i.e., individuals with TRD). Third, we incorporated parameter uncertainty through probabilistic analyses, which allowed us to measure the impact of dynamic changes in model inputs. Finally, our study explored the cost-effectiveness of PAT in the US context, which has different cost considerations than the UK.

Our sensitivity analyses suggest that improvements in the effectiveness of PAT—measured in terms of its ability to promote and sustain remission—can increase the probability that PAT is cost-effective compared to SOC. However, we also found that the cost of PAT is likely to be the most influential factor when assessing its economic value. The cost of psychological support can be reduced by offering group therapy [64], allowing therapists to monitor simultaneous sessions [65], and utilizing alternative clinical personnel [66]. However, the efficacy of these types and formats of psychological support requires further testing. The price of the psilocybin itself is also a significant portion of the total PAT cost, and a wide range of factors will affect the final price. These include risk mitigation requirements from the FDA, patents on psilocybin analogs (which is opposed by some groups on ethical, legal, and sociocultural grounds [67, 68]), effective demand, competition from other effective therapies (including other psychedelics), and sensitivity of third-party payers to these costs.

We used limited healthcare and societal perspectives in this analysis, meaning we omitted costs and outcomes that may influence PAT’s short- and long-term value. For example, we excluded caregiving, transportation, and patient time costs in this analysis, which are included in a full societal perspective analysis [51]. If patients with TRD spend more time receiving PAT than alternative MDD treatments, then PAT’s incremental costs will increase, and its probability of being a cost-effective treatment will decrease. Another important outcome that we excluded is caregiver burden: research has shown that caregivers of patients with MDD are more likely to develop MDD themselves, experience financial strain, and report lower health-related quality of life [69, 70]. The effect of PAT on caregiver burden is unknown and requires investigation.

Economic evaluations like this study are contributing to the rapidly evolving field of psychedelic science [59]. Prior analyses, such as those on MDMA-assisted therapy [71–73], have highlighted the importance of assessing the economic value of novel technologies that are more effective but costlier than usual care. PAT can also benefit from similar economic evaluations as more evidence about its costs and effectiveness becomes available to ensure that PAT remains efficient, affordable, and equitably accessible. Concerns about affordability and accessibility are especially salient for MDD and TRD, given evidence that populations experiencing different vulnerabilities, such as individuals who are low-income, unemployed, and have substance use disorders, are more likely to experience these conditions.[74–77] Research has also documented significant disparities in access to MDD and TRD treatments. For example, while racial and ethnic minorities are prescribed depression treatments as frequently as White patients, they are less likely to take antidepressants or receive specialty mental healthcare [78–80]. Among people with TRD, those low socioeconomic status are less likely to receive novel treatments like esketamine [81]. Future studies can use a distributional cost-effectiveness framework [82] to understand PAT’s cost-effectiveness and impact on equity while assuming different levels of access and acceptance of this treatment across key subgroups.

### Limitations

This study has several limitations. The time horizon was set to a year due to the lack of long-term data on the effectiveness of PAT. Future analyses should use longer time horizons when data becomes available. We excluded some therapies for TRD (e.g., repetitive transcranial magnetic stimulation, intravenous ketamine therapy), and their inclusion will likely affect PAT’s overall and relative economic value. We narrowly assessed the cost-effectiveness of one-time, single-dose PAT as a third-line therapy for TRD. Several trials have demonstrated the efficacy of PAT as treatment for acute MDD episodes [14, 15, 20, 83], and future analyses can explore PAT as a different line of therapy or as maintenance or continuation therapy. We assumed that all simulated individuals received PAT instead of SOC, which is supported by recent survey data suggesting that most US adults with anxiety, depression, or PTSD who receive medication for their condition are open to exploring treatments such as ketamine (66%), psilocybin (62%), and MDMA (56%) [84]. Similar levels of acceptance of psilocybin and other psychedelic therapies have been documented among diverse populations, including college students, mental health professionals, and racial minorities with a substance use disorder in the US; mental health service users in Ireland; and individuals with experiences with childhood trauma in Canada [85–88]. However, factors like stigma, legal barriers, health risks, and PAT’s novelty may still discourage people from seeking or receiving PAT [89, 90]. Future studies should incorporate treatment uptake as a model parameter. The level of uncertainty around several model parameters is not well characterized in the literature, and we relied on the best-available evidence or reasonable assumptions where needed. For example, while our model simulated individuals, only all-cause mortality rates varied by age and gender, and most inputs, including the effectiveness of different MDD treatments, were assumed to be uniform across subgroups. Additional work is needed to improve model parameter estimates, such as ethno-racial differences in psychedelic psychopharmacology [91]. We used 2020 mortality rates in our model, the most recent available data when we conducted our study, which may be higher than current mortality rates due to the COVID-19 pandemic. Our probabilistic model, however, incorporated the uncertainty around mortality due to MDD and other causes.

## Conclusion

PAT is an effective treatment for MDD and TRD, and it has the potential to be a cost-effective therapy under specific conditions. PAT can be a cost-effective option for TRD compared to standard treatments if costs are $5000 or lower. Future trials should explore ways the cost of PAT can be reduced.

## Supplementary information

Supplement 1

Supplement 2

## Data Availability

All model inputs used in this cost-effectiveness analysis are fully reported within the manuscript. The model itself is available from the corresponding authors upon reasonable request.
