# Supplementary material for: Psilocybin-assisted therapy for treatment-resistant depression in the US: a model-based cost-effectiveness analysis
Source: Transl Psychiatry. 2025 Aug 29;15:330. doi: 10.1038/s41398-025-03556-4 (PMC12397412; doi:10.1038/s41398-025-03556-4)
Supplement: Supplementary file 2 — Supplement 2 [file 41398_2025_3556_MOESM2_ESM.docx]

# S2. Appendix

[Table A1. Recommended treatments for major depressive disorder](#_Toc174032257)

[Table A2. Impact Inventory](#_Toc174032258)

[Table A3. Additional cost inputs](#_Toc174032259)

[Figure A1. Cost-effectiveness acceptability curves (A) and expected loss curves (B) using a limited healthcare perspective and $10,000 cost of PAT](#_Toc174032260)

[Figure A2. Cost-effectiveness acceptability curves (A) and expected loss curves (B) using a limited societal perspective and $5,000 cost of PAT](#_Toc174032261)

[Figure A3. Cost-effectiveness acceptability curves (A) and expected loss curves (B) using a limited societal perspective and $10,000 cost of PAT](#_Toc174032262)

[Figure A4. Cost-effectiveness acceptability curves assuming $3,000 cost of PAT using limited healthcare (A) and limited societal (B) perspectives.](#_Toc174032263)

[Figure A5. Cost-effectiveness acceptability curves assuming $15,000 cost of PAT using limited healthcare (A) and limited societal (B) perspectives.](#_Toc174032264)

[Figure A6. Cost-effectiveness acceptability curves assuming $20,000 cost of PAT using limited healthcare (A) and limited societal (B) perspectives.](#_Toc174032265)

## Table A1. Recommended treatments for major depressive disorder

| **Line of therapy** | **Type** | **Treatment** | **Estimated prevalence** |
| --- | --- | --- | --- |
| 1^a^ | Pharmacotherapy | SSRI (Citalopram, Fluoxetine, Paroxetine Escitalopram, Sertraline, Fluvoxamine), SNRIs (venlafaxine XR, venlafaxine IR, desvenlafaxine, duloxetine) | 100% |
|  | Psychotherapy | Behavioral therapy, cognitive therapy, CBT, mindfulness-based cognitive therapy, interpersonal psychotherapy, psychodynamic therapies, and supportive therapy^1^ |  |
| 2^a^ | Pharmacotherapy | SSRIs (citalopram, sertraline), SNRI (venlafaxine XR, venlafaxine IR, desvenlafaxine, duloxetine), anxiolytic (buspirone), NDRI (bupropion SR) | 100% |
|  | Psychotherapy | CBT and cognitive therapy (combined with pharmacotherapy) |  |
| 3 | Pharmacotherapy | Cyclic antidepressants (nortriptyline, mirtazapine), lithium augmentation, T3 augmentation (dothyronine) | 99.59% |
|  |  | Esketamine | 0.16%^c^ |
|  | Medical | ECT | 0.25%^2^ |
| ≥4 | Pharmacotherapy | MAOIs (selegiline, isocarboxzaid, phenelzine, tranylcypromine, moclobemide), SNRI (venlafaxine XR), cyclic antidepressants (mirtazapine) | 100% |

^a^First and second lines of therapy for MDD were not included in this analysis.

^b^We did not include some antidepressant treatments such as (rTMS), which lack prevalence data, and vagus nerve stimulation (VNS), which is recommended only for long-lasting treatment-resistant depression and beyond the 12-month time horizon of our analysis.

^c^Estimated using the total number of esketamine users (30,000) as of November 2022 and the number of individuals with TRD in the US (6.3 million).

CBT, cognitive behavioral therapy; ECT, electroconvulsive therapy; NDRI, norepinephrine and dopamine reuptake inhibitors; SNRI, serotonin and norepinephrine reuptake inhibitors; SSRI, selective serotonin-reuptake inhibitor.

## Table A2. Impact Inventory

| **Sector** | **Type of Impact** | **Limited healthcare sector** | **Limited societal** | **Notes on sources of evidence** |
| --- | --- | --- | --- | --- |
| **Formal healthcare sector** | | | | |
| **Health** | Longevity |  |  | Excluded due to limited time horizon |
|  | Health-related quality-of-life effects | X | X | Health utilities from Sapin et al. 2004 (Table 1) |
|  | Other health effects (e.g., caregiver health-related quality of life) |  |  | Excluded |
|  | Medical costs paid for by third-party payers | X | X | Amos et al. 2018; Agbese et al. 2022; Ross et al. 2018; Brendle et al. 2022 (Table 1) |
|  | Medical costs paid for by patients out-of-pocket |  |  | Excluded |
|  | Future related medical costs |  |  | Excluded due to limited time horizon |
|  | Future unrelated medical costs |  |  | Excluded due to limited time horizon |
| **Informal healthcare sector** | | | | |
| Health | Patient costs |  |  | Excluded |
|  | Unpaid caregiver time costs |  |  | Excluded |
|  | Transportation costs |  |  | Excluded |
| **Non-healthcare sectors** | | | | |
| Productivity | Formal labor market earnings lost |  | X | Zhdanava et al. 2021 (Table 1) |
|  | Cost of unpaid lost productivity due to illness |  | X | Zhdanava et al. 2021 (Table 1) |
|  | Cost of uncompensated household production |  |  | Excluded |
| Consumption | Future consumption unrelated to health |  |  | Excluded |
| Social services | Costs associated with social welfare services |  |  |  |
| Legal/criminal justice | Costs associated with the justice system, police protection, judicial and legal costs, and corrections |  |  | Excluded |
| Education | Impact of intervention on educational achievement of population |  |  | Excluded |
| Housing | None |  |  | Excluded |
| Environment | None |  |  | Excluded |

## Table A3. Additional cost inputs

| **Line of therapy** | **Pharmacotherapy costs**^4^ | | **Healthcare costs of MDD**^4^ | | **Healthcare costs of response**^4^ | | **Productivity cost of MDD**^8^ | |
| --- | --- | --- | --- | --- | --- | --- | --- | --- |
|  | *Base value (SD)* | *Range* | *Base value (SD)* | *Range* | *Base value (SD)* | *Range* | *Base value (SD)* | *Range* |
| 4 | 13 (0.64) | 11-14 | 237 (12) | 213-261 | 118 (6) | 107-130 | 155 (36) | 84-226 |
| 5 | 14 (0.72) | 13-15 | 245 (12) | 220-269 | 118 (6) | 107-130 | 155 (36) | 84-226 |
| 6 | 16 (0.79) | 14-17 | 284 (14) | 256-313 | 118 (6) | 107-130 | 155 (36) | 84-226 |
| ≥7 | 19 (0.97) | 17-21 | 330 (17) | 297-363 | 118 (6) | 107-130 | 155 (36) | 84-226 |

MDD, major depressive disorder; SD, standard deviation.

## Figure A1. Cost-effectiveness acceptability curves (A) and expected loss curves (B) using a limited healthcare perspective and $10,000 cost of PAT

| 1. **Cost-effectiveness acceptability curves**    |
| --- |
| 1. **Expected loss curves**    |

The graphs in this figure summarize the results of the probabilistic analyses. Cost-effectiveness acceptability curves (A) display the probability that each intervention is cost-effective over a range of cost-effectiveness thresholds. The probabilities are calculated based on the results of the 1,000 simulations. Expected loss curves (B) plot the per-person cost or forgone benefits of choosing a suboptimal intervention given current evidence. The intervention with the lowest expected loss at any cost-effectiveness threshold is the optimal choice. The cost-effectiveness threshold where the curves cross is equal to the expected incremental cost-effectiveness ratio of PAT. The gray horizontal band represents the conventional cost-effectiveness threshold range used in the US ($100,000-150,000 cost per QALY gained).

PAT, psilocybin-assisted therapy; QALY, quality-adjusted life year.

## Figure A2. Cost-effectiveness acceptability curves (A) and expected loss curves (B) using a limited societal perspective and $5,000 cost of PAT

| 1. **Cost-effectiveness acceptability curves**    |
| --- |
| 1. **Expected loss curves**    |

The graphs in this figure summarize the results of the probabilistic analyses. Cost-effectiveness acceptability curves (A) display the probability that each intervention is cost-effective over a range of cost-effectiveness thresholds. The probabilities are calculated based on the results of the 1,000 simulations. Expected loss curves (B) plot the per-person cost or forgone benefits of choosing a suboptimal intervention given current evidence. The intervention with the lowest expected loss at any cost-effectiveness threshold is the optimal choice. The cost-effectiveness threshold where the curves cross is equal to the expected incremental cost-effectiveness ratio of PAT. The gray horizontal band represents the conventional cost-effectiveness threshold range used in the US ($100,000-150,000 cost per QALY gained).

PAT, psilocybin-assisted therapy; QALY, quality-adjusted life year.

## Figure A3. Cost-effectiveness acceptability curves (A) and expected loss curves (B) using a limited societal perspective and $10,000 cost of PAT

| 1. **Cost-effectiveness acceptability curves**    |
| --- |
| 1. **Expected loss curves**    |

The graphs in this figure summarize the results of the probabilistic analyses. Cost-effectiveness acceptability curves (A) display the probability that each intervention is cost-effective over a range of cost-effectiveness thresholds. The probabilities are calculated based on the results of the 1,000 simulations. Expected loss curves (B) plot the per-person cost or forgone benefits of choosing a suboptimal intervention given current evidence. The intervention with the lowest expected loss at any cost-effectiveness threshold is the optimal choice. The cost-effectiveness threshold where the curves cross is equal to the expected incremental cost-effectiveness ratio of PAT. The gray horizontal band represents the conventional cost-effectiveness threshold range used in the US ($100,000-150,000 cost per QALY gained).

PAT, psilocybin-assisted therapy; QALY, quality-adjusted life year.

## Figure A4. Cost-effectiveness acceptability curves assuming $3,000 cost of PAT using limited healthcare (A) and limited societal (B) perspectives.

| 1. **Limited healthcare perspective**    |
| --- |
| 1. **Limited societal perspective**    |

Cost-effectiveness acceptability curves (A) display the probability that each intervention is cost-effective over a range of cost-effectiveness thresholds. The probabilities are calculated based on the results of the 1,000 simulations.

PAT, psilocybin-assisted therapy; QALY, quality-adjusted life year.

## Figure A5. Cost-effectiveness acceptability curves assuming $15,000 cost of PAT using limited healthcare (A) and limited societal (B) perspectives.

| 1. **Limited healthcare perspective**    |
| --- |
| 1. **Limited societal perspective**    |

Cost-effectiveness acceptability curves (A) display the probability that each intervention is cost-effective over a range of cost-effectiveness thresholds. The probabilities are calculated based on the results of the 1,000 simulations.

PAT, psilocybin-assisted therapy; QALY, quality-adjusted life year.

## Figure A6. Cost-effectiveness acceptability curves assuming $20,000 cost of PAT using limited healthcare (A) and limited societal (B) perspectives.

| 1. **Limited healthcare perspective**    |
| --- |
| 1. **Limited societal perspective**    |

Cost-effectiveness acceptability curves (A) display the probability that each intervention is cost-effective over a range of cost-effectiveness thresholds. The probabilities are calculated based on the results of the 1,000 simulations.

PAT, psilocybin-assisted therapy; QALY, quality-adjusted life year.
